# Fabry-Perot Interference Fiber Acoustic Wave Sensor Based on Laser Welding All-Silica Glass

**DOI:** 10.3390/ma15072484

**Published:** 2022-03-28

**Authors:** Wenhua Wang

**Affiliations:** School of Electronic and Information Engineering, Guangdong Ocean University, Zhanjiang 524088, China; wangwh@gdou.edu.cn

**Keywords:** optical fiber acoustic wave sensor, sensor probe, Fabry–Perot interferometer, laser welding, silica glass

## Abstract

Due to the small difference between the thermal expansion coefficients of silica optical fiber and silica glass, they are used as probe materials of optical fiber acoustic wave sensors. According to the light absorption characteristics of a pressure-sensitive silica diaphragm and silica glass, the laser welding of an all-silica Fabry–Perot (FP) interference optical fiber acoustic wave sensor with a CO_2_ laser is proposed. For understanding the influence of thermal expansion of sealing air in an FP cavity and the drift of interference-intensity demodulation working point of a FP interference acoustic wave sensor, we designed a process for the laser welding of an ultra-thin silica diaphragm and sleeve and optical fiber and sleeve. The exhaust hole of the FP cavity is reserved in the preparation process, and an amplified spontaneous emission light source and a tunable optical-fiber FP filter are introduced to stabilize the working point. The sensor is tested with a 40 kHz sound vibration signal. The results show that the sound pressure sensitivity of the sensor to an acoustic source of 0.02–0.1 W/cm^2^ is 6.59 mV/kPa. The linearity coefficient is 0.99975, indicating good linearity.

## 1. Introduction

There has been an increase in the demand for sensors in recent decades owing to societal developments and an increase in the use of artificial intelligence. However, electronic sensors have disadvantages due to electrical signals, such as sensitivity to electromagnetic signals, fast attenuation, and inconvenience for multiplexing. In contrast, optical-fiber sensors are not susceptible to electromagnetic interferences, have good insulation, are thin, exhibit high resolutions, allow long-distance transmissions, and can be easily reused. Hence, in the past few decades, they have been widely investigated and used in structural health, industrial, aerospace architecture, medical, and environmental monitoring [1,2,3,4,5,6,7,8,9]. Moreover, optical-fiber sensing technology has been used for acoustic signal detection in various fields as well [2,8,10,11,12,13,14]. Among them, the partial discharge monitoring and localization of power transformer is an important application of this kind of sensor. The partial discharge of power transformer may cause major disasters, and its acoustic emission frequency is tens to hundreds of kHz. The frequency of 40–300 kHz should be selected for ultrasonic diagnosis of partial discharge of the transformer [15]. The optical fiber acoustic sensor has become a research hotspot in this field because of its unique advantages. As early as 1977, Nelson et al. [16] used an optical fiber bent into a “U” shape and fixed it to a film at the bending position. The film was deformed using an acoustic signal, which in turn slightly bent the optical fiber, and the acoustic signal was detected. In 1991, Garthe [17] built an acoustic signal detection device with a single-mode fiber, reflective film, and self-focusing lens. Presently, intensity modulation and interference optical-fiber acoustic wave sensors are mainly used in various fields, and the development of optical-fiber technology has highlighted the advantages of these sensors in different real-life applications. The interference optical-fiber acoustic wave sensors can be realized using fiber Mach–Zehnder (MZ), Michelson (M), and Fabry-Perot (FP) interferometers.

In the MZ and M interference optical-fiber acoustic wave sensors, the elastic-optical effect of the optical fiber is negligible, and thus, an extremely long optical fiber must be used in these interference sensors to obtain a high sensitivity. However, using a long fiber causes poor thermal stability and sensitivity to vibration. In addition, the phase noise caused by the light source has a significant influence on the interferometer structure, which requires a highly coherent light source for adequate sensor performance. Moreover, there are stringent requirements on the polarization state of the optical signal during the optical-fiber transmission. However, such requirements are difficult to fulfill in practical applications, because the birefringence of the optical fiber causes random changes in the polarization state of the optical signal during the optical fiber transmission. Therefore, these sensors exhibit polarization fading [18]; that is, the randomly changing polarization state in the optical fiber reduces the interference fringe contrast. Thus, FP interference fiber-optic acoustic wave sensors, especially the FP interference fiber-optic sensors based on diaphragm deformation, have attracted the attention of many researchers [14]. The optical-fiber sleeve, diaphragm material, and the structure and manufacturing process of the sensor are the main factors that can be changed to improve its performance. Xu et al. [19,20] and Pulliam et al. [21] proposed an FP sensor with an SiC diaphragm [20] to improve the sensor performance. In 2013, Ma et al. [22] realized an acoustic wave sensor with a graphene pressure-sensitive diaphragm that can respond to frequencies in the range of 0.2–22 kHz. In 2017, Gong et al. [23] proposed an ultra-low-voltage acoustic wave field sensor with a parylene diaphragm and stainless steel sleeve. In 2018, Ni et al. [24] proposed an FP interference fiber acoustic wave sensor with an ultra-thin graphene diaphragm. In 2020, Qi et al. [25] and Zhang et al. [26] proposed flywheel and gold diaphragm-based FP interference fiber acoustic wave sensors, respectively, and achieved a good sensor performance. Using different ferrule and diaphragm materials can improve the performance of the sensor to a certain extent. However, owing to the differences in the thermal expansion coefficients of materials, the ferrule and diaphragm may be squeezed owing to environmental impact when the sensor is used, thus affecting its performance. In addition, FP cavities are often sealed, which causes unnecessary temperature–sound pressure cross sensitivity due to the thermal expansion effect of the residual air sealed in the cavity, decreasing the measurement accuracy. This problem can be solved by using laser drilling [20], through fabrication in vacuum [27], or using a temperature compensation scheme [28]; however, these techniques increase the complexity of the process and system.

As everyone knows, the material of optical fiber is silica glass, so the ferrule is usually made of silica glass. Therefore, the glass membrane is one of the best schemes, and researchers employs different methods to realize this kind of sensor with glass diaphragm. In this study, a silica optical fiber, silica sleeve, and an ultra-thin silica glass pressure-sensitive diaphragm were used to construct an FP interference optical-fiber acoustic wave sensor made of all-silica materials, and an all-laser welding preparation method was adopted to ensure that the FP cavity can be easily opened during the fabrication process. The all-silica material mitigates the shortcomings caused by the differences in the thermal expansion of materials as well as those arising from sensor aging or high-temperature-induced failure because of gluing.

## 2. Structure and Fabrication of Sensor

### 2.1. Structure of the Sensor

**Figure 1:**
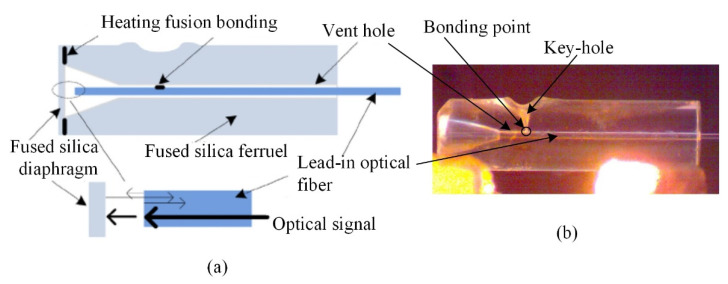
Structure of the sensor. (**a**) schematic diagram, (**b**) physical photo.

The structure of the sensor is shown in Figure 1a. Figure 1b shows the structure of the welded object. The silica glass sleeve had a length of 7 mm, an outer diameter of 1.8 mm, and an inner diameter in the range of 126–128 μm through hole. One end of the sleeve had a bell mouth with an inner diameter of 1 mm. The thickness of the ultra-thin silica glass pressure-sensitive diaphragm was 30 μm; its inner surface was polished to reflect the light signals, and its outer surface was frosted. A single-mode optical fiber was used, and the end face of the optical fiber and the inner surface of the diaphragm formed an FP cavity with a length (*L*) of 49.92 μm. The gap left in the optical fiber through a hole was used as the vent hole of the FP cavity to avoid the influence of thermal expansion of the residual air, present in the closed FP cavity, on the sensor.

### 2.2. Fabrication of Sensor

**Figure 2:**
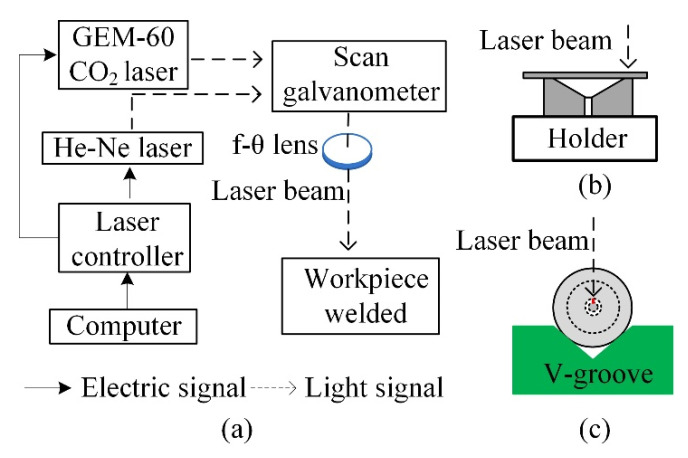
Welding optical path system. (**a**) schematic block diagram; (**b**) fixture of diaphragm; and (**c**) fixture of ferrule.

**Figure 3:**
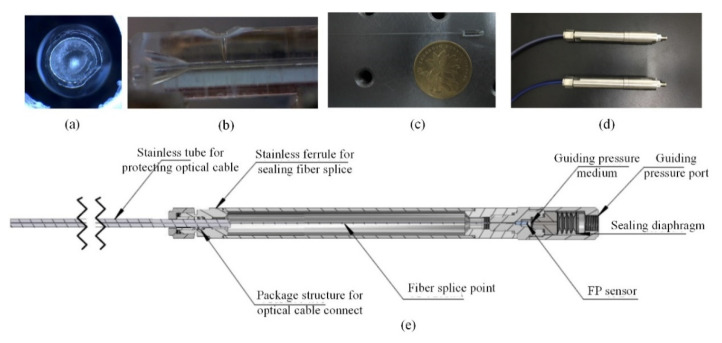
Laser-welded sensor head and sensor package. (**a**) physical photo of diaphragm after welded; (**b**) physical photo of ferrule after welded; (**c**) physical photo of welded sensor without package; (**d**) physical photo of packaged sensor; and (**e**) schematic diagram of sensor package.

The sensor was fabricated using all-laser welding. The welding optical path system is shown in Figure 2a, where a CO_2_ laser was the welding light source, and an He–Ne laser was used to adjust the optical path. First, the end face of the bell mouth of the sleeve was polished via grinding; its end face was perpendicular to the axis of the sleeve. The CO_2_ laser is pulsed laser with a frequency of 24 kHz. The optimal parameter for welding is determined by real-time monitoring the temperature of the welding area during welding with different parameters by the noncontact infrared radiation thermometer (ISQ5-LO, Lumasense Inc., Frankfurt, Germany). According to the temperature measurement results and the softening temperature of the silica-glass, the laser with a duty cycle of 60% is employed to rotate for 40 cycles at the speed of 600 r/min for welding of a diaphragm; the laser with 80% duty cycle is used to irradiate the welding point for 0.9 s for welding of an optical fiber. After cleaning and drying, it was fixed on the fixture as shown in Figure 2b. The processed diaphragm was slightly glued on the end face of the bell mouth and then placed under the laser beam. The fixture was rotated using a stepping motor to seal the diaphragm and end face of the sleeve via welding. Second, the ferrule, welded with the diaphragm, was placed on the fixture as shown in Figure 2c, and the processed optical fiber was inserted into through the hole of the ferrule until the cavity length *L* was 49.92 μm. The adjusting frame of the fixture was adjusted until the laser beam was aligned with the designed welding point to realize single-point welding for fixing the ferrule and optical fiber. During the welding and fixing of the optical fiber, an exhaust hole was reserved for the FP cavity to ensure the formation of an open cavity and improve the performance of the sensor. The laser-welded sensor head along with the sensor packaging are shown in Figure 3. Figure 3a shows the top view of the pressure sensor diaphragm after welding, and Figure 3b illustrates the welding of the optical fiber and silica sleeve. Figure 3c,d depict the outlines of the welded sensor head and sensor package. The temperature–pressure cross sensitivity of the laser-welded open-cavity FP interference fiber sensor is extremely low, i.e., approximately 0.025 nm/°C [29]. This type of sensors prepared by other fabrication methods have relatively high temperature-pressure cross sensitivity. For example, the sensor in reference [30] is 0.29 nm/°C and the sensor in reference [31] is 0.28 nm/°C and 3.38 nm/°C.

## 3. Principle and Signal Demodulation of the Sensor

As explained before, the circular diaphragm was fixed on the end face of the sleeve via laser welding. When the acoustic signal acts on the diaphragm, it deforms the diaphragm, which changes the cavity length of the FP cavity. For the central position of the diaphragm, where the optical fiber is aligned, the deformation *Y* can be expressed as [32]

(1) $$Y=\frac{3(1-\mu^{2})P}{16Eh^{3}}a^{4}$$

wherein *μ*, *E*, *h*, and *a* represent the Poisson’s ratio, Young’s modulus, thickness and semidiameter of the sensor diaphragm. The parameters of silica glass are Poisson’s ratio of 0.17 and Young’s modulus of 73 GPa. The diaphragm parameters can be selected according to application requirements. In this paper, the parameters of the diaphragm are 30 μm in thickness and 0.5 mm in semidiameter.

**Figure 4:**
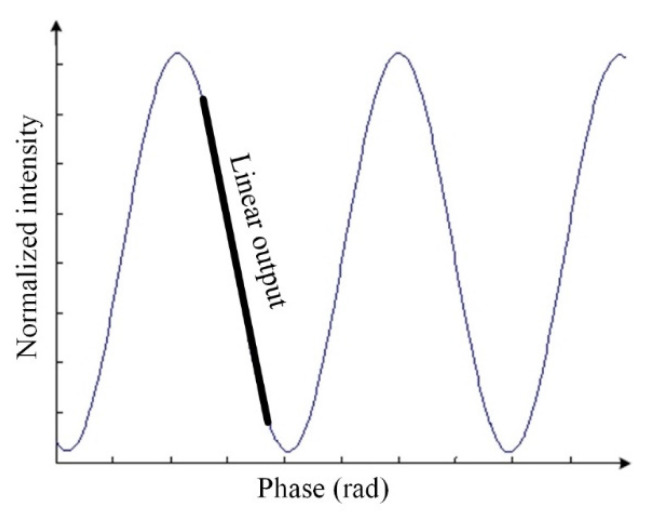
Working area of the interference-intensity demodulation.

**Figure 5:**
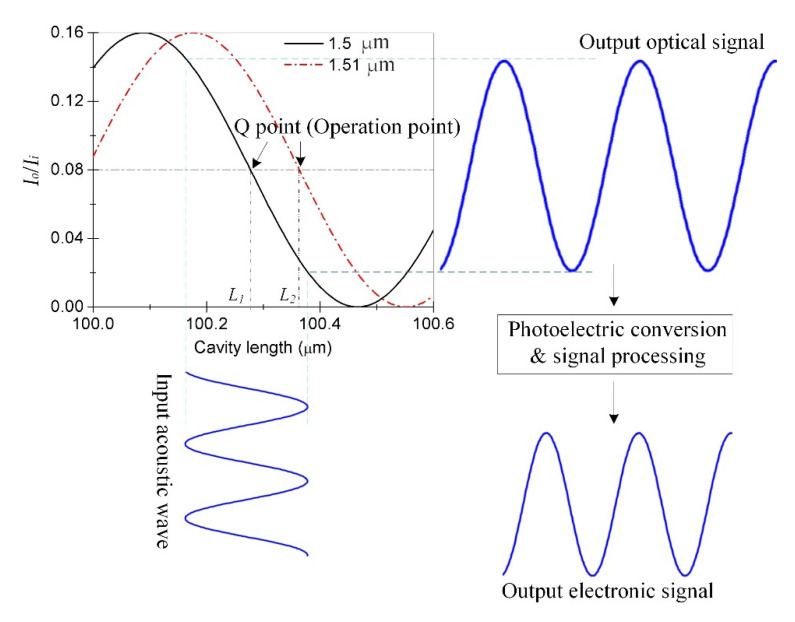
Formation process of the output signal of the acoustic waver sensor.

For the detection of acoustic signals in FP interference sensors, coherence-intensity demodulation is typically implemented because of a simple demodulation process. When the acoustic wave signal acts on the diaphragm to produce a slight deformation, if the deformation of the cavity length *L* is within *λ*/4, then the change in the interference fringes will be within half period of that of the interference signal. The change in the cavity length corresponds to the change in light intensity, and the direction of its change can be determined, as shown by the thick line in Figure 4. Therefore, after determining the appropriate the *L* of sensor and light source wavelength, if the change of *L* caused by sound pressure is within half a period of interference fringe, the periodicity between the cavity length change and the output signal becomes linear. Based on this, the sound pressure change is set in a certain range during the subsequent sensor test in this paper. As evident from Figure 5, the output signal of the FP interference fiber acoustic wave sensor is formed when the O point (operation point) of the sensor is set at the Q point (quadrature point) of the FP interference signal such that the change in light intensity caused by the change in the interference fringes has a linear relationship with the change in the cavity length; any deviation from this linearity causes performance degradation in the sensor. Therefore, the stability of the O point is important for FP interference fiber-optic acoustic wave sensors. That is, the change in the cavity length caused by a non-acoustic signal must be compensated by a change in the incident light wavelength to ensure that the O point can be stabilized at the Q point in environmental monitoring applications. The real and imaginary interference curves in the figure are the cavity lengths *L*_1_ and *L*_2_ with the wavelengths of 1.5 and 1.51 μm, respectively. For the condition when *L*_1_ becomes *L*_2_ because of changes in the non-acoustic signal in the environment, if the wavelength of the light source is adjusted from 1.50 to 1.51 μm, then the O point can be stabilized at the Q point during the detection of the dynamic acoustic signal by the sensor such that the performance of the sensor remains unaffected.

## 4. Working-Point Stabilization of the Sensor

**Figure 6:**
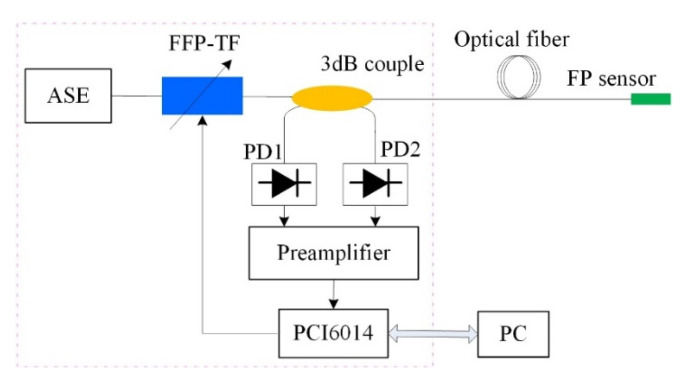
Operating point self-stabilizing system based on an ASE light source and tunable optical-fiber FP filter.

In this study, an amplified spontaneous emission (ASE) light source with a wavelength of approximately 1530–1570 nm was used, and a fiber FP filter (Micron Optics Inc., Atlanta, TX, USA) was used to realize the specific working wavelength of the incident FP acoustic wave sensor. The filter was also used to change the working wavelength to compensate for the change in the cavity length caused by the non-acoustic signals in the environment. The working point self-stabilizing system is shown in Figure 6, the sensor’s working-point stabilization system consists of an ASE broadband light source, a fiber FP tunable filter, a 3-dB fiber coupler, a photodetector, preamplifier and a data acquisition card (PCI6014, NI Corporation, Austin, TX, USA). The optical signal emitted by the ASE light source is the output of the tunable fiber FP filter with the required working wavelength that corresponds to the Q point of the initial cavity length. The signal generated after the source-emitted signal passes through the fiber coupler is directly converted into an electrical signal by the PD_2_ detector and transmitted to the FP cavity of the sensor. The acoustic signal acts on the pressure-sensitive element of the FP cavity, such that the modulated optical signal returns to point Q and is converted into an electrical signal by the PD1_1_ detector after passing through the fiber coupler. To eliminate the influence of the fluctuation of the light source on the acoustic signal detection, when processing the received electrical signals, the electrical signals output by PD_1_ and PD_2_ are compared for subsequent processing.

The relationship between the change in the working wavelength (*λ*) and that in the the cavity length (*L*) can be expressed as follows:

(2) $$\Delta L=\frac{L}{\lambda }\Delta \lambda$$

**Figure 7:**
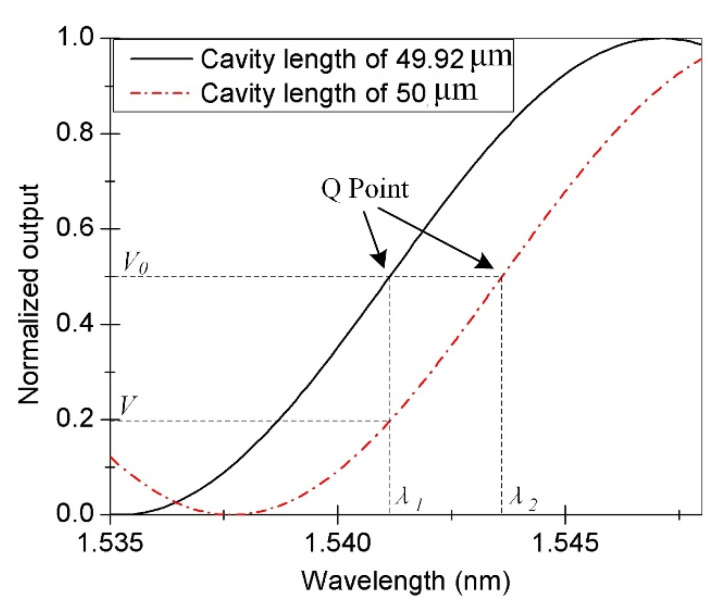
Working-point wavelength corresponding to the Q point under different cavity lengths.

According to the cavity length, working wavelength of the sensor, and characteristics of the interference spectrum, we consider the interference spectrum in the wavelength range of 1535 to 1548 nm, as shown by the solid line in Figure 7. According to the characteristics of the Q point, the curve derivative is used to find the maximum value. The position corresponding to this value is the Q point, whose output voltage signal is determined as *V*_0_, and the corresponding driving voltage of the tunable fiber FP filter is *U*_0_. When the acoustic wave sensor is used in practice, the output voltage signal *V* corresponding to the working point of the sensor can be obtained using a low-pass filter. If the FP cavity length changes because of the influence of non-acoustic signals in the environment, then *V* deviates from *V*_0_, which corresponds to the Q point of the original cavity length. If the cavity length increases by 0.08 μm owing to the influence of the non-acoustic signals, then the interference spectrum shown by the dotted line in Figure 7 is obtained. To ensure that the performance of the sensor is not affected, the driving voltage U of the tunable filter must be adjusted, such that its output wavelength changes, and the working point of the sensor returns to the Q point. In the figure, *λ*_1_ and *λ*_2_ are the wavelengths corresponding to the Q point before and after the cavity length changes, respectively.

## 5. Experimental Results and Analysis

### 5.1. Acoustic Wave Signal Measurement

**Figure 8:**
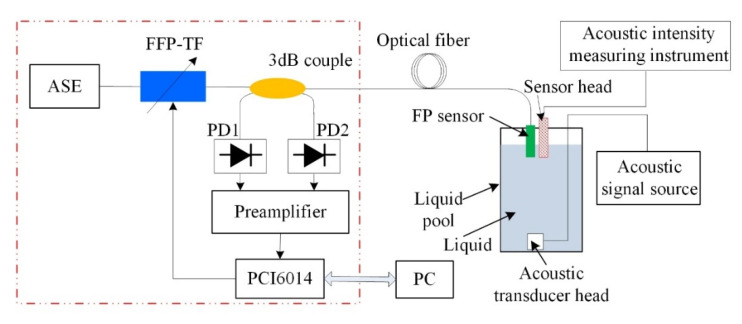
Schematic of the ultrasonic signal testing system.

The sound wave signal measuring device is shown in Figure 8. The sound intensity measuring instrument is used to measure the sound pressure sensitivity for Section 5.2. The probe of the FP interference fiber acoustic waver sensor was placed in a liquid pool, and the acoustic signal transmitter was placed at the bottom of the pool. The acoustic signal transmitter transmitted a 40 kHz acoustic signal to the FP interference sensor. The tunable optical-fiber FP filter forms the working wavelength corresponding to the Q point of the sensor FP cavity under the action of the working-point stabilization system. During the experiment, the working wavelength was determined by changing the position of PD_2_ to the optical-fiber spectrometer display. According to the cavity length parameter of the sensor, one quarter of the periodic signal was selected as the working range; hence, the working wavelength was determined to be 1541 nm.

**Figure 9:**
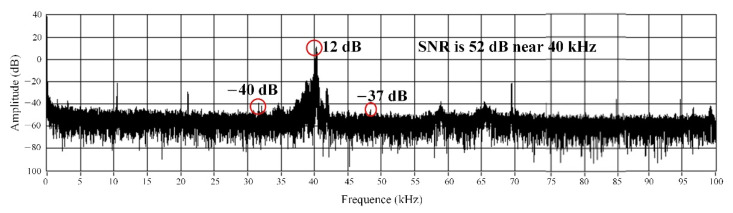
Frequency-domain signal of the sensor.

**Figure 10:**
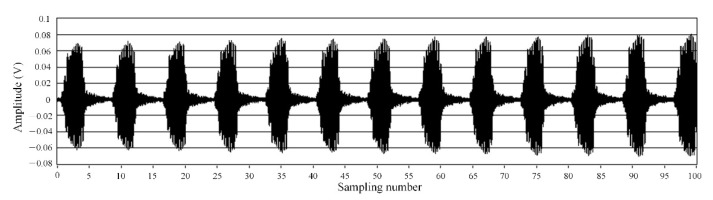
Time-domain signal of the sensor.

The sound pressure acting on the pressure-sensitive element of the sensor was provided by the output power of the acoustic signal source, with the frequency of 40 kHz. The silica glass diaphragm is deformed under the sound pressure. The sensor outputs the signal according to the principle shown in Figure 5, and its frequency-domain signal is shown in Figure 9. The results show that the amplitude was maximized at the frequency of 40 kHz, and there was almost no other signal with an amplitude. In Figure 9, the background is high as well as the noise, which may be due to the poor signal receiving circuit made by ourselves, and the noise filtering by the filter circuit is not very good. In addition, when the ultrasonic signal generator works, its support rod will vibrate slightly. The vibration signal causes the FP sensor to receive additional signals that are not filtered out. However, as can be seen from Figure 9, near the frequency with 40 kHz of the ultrasonic generator, the signal-to-noise ratio is as high as about 52 dB, and the influence of background and noise on signal recognition is insignificant. In addition, the time-domain spectrum waveform corresponding to the frequency-domain signal in Figure 9 is regular, and its peak-to-peak value is approximately 130 mV shown in Figure 10. From Figure 10, there is basically no noise in the time-domain waveform.

### 5.2. Sound Pressure Sensitivity of the Sensor

**Figure 11:**
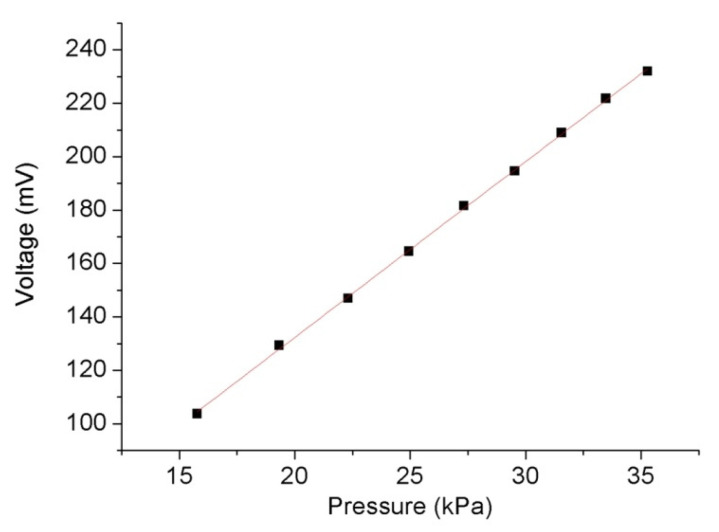
Sensitivity of the sound pressure-output voltage of the FP interference fiber-optic acoustic waver sensor.

The sensitivity test system is shown in Figure 8. The sound intensity of the FP interference fiber acoustic waver senso probe is less than that detected by the sound intensity detector; hence, the sound intensity detector and sensor probes were placed in the same position. The sound pressure received by the pressure sensor was calculated according to the sound intensity received by the detector, and the relationship between the sound pressure *P* and sound intensity *I* is expressed as follows:

(3) $$P_{}^{2}=I\rho v$$

where *ρ* is the density of the liquid in the liquid pool, which was 877 kg/m^3^ for the transformer oil used in this experiment, υ is the propagation speed of the acoustic signals in the transformer oil, and the theoretical value at 25 °C, that is, 1420 m/s, was used in this calculation. According to the third section of this paper, coherent-intensity demodulation requires the acoustic waver sensor to work in the linear region of the interference spectrum; hence, it can be seen from Figure 4 that the maximum change in the cavity length due to the acoustic signal vibration should be less than 1/4 (approximately 382 nm) or 1/6 (approximately 256 nm) of the working wavelength. According to Equation (1) and the parameters of the silica diaphragm pressure sensor, the sensitivity of the change in the cavity length △*L* with the change in pressure change was approximately 5.77 nm/kPa. In this study, the output power of the acoustic signal source was adjusted to 0.02–0.1 W/cm^2^, and the corresponding pressure of the acoustic signal was 15.78–35.29 kPa. During the test, there was a test point after each 0.01 W/cm^2^, and the output voltage of the sensor corresponding to the sound intensity and pressure was recorded at the test point. The results shown in Figure 11 suggest that the all-silica FP interference fiber acoustic wave sensor prepared using laser welding has good linearity, with a linear correlation coefficient *R* of 0.99975 and sensitivity of 6.59 mV/kPa.

## 6. Conclusions

Temperature-pressure cross sensitivity and operating point drift are the key problem of this kind of sensor, which affects the practical application of the sensor. Researchers have been trying to better solve these problems in recent 20 years. An all-laser welding technique was employed to fabricate the FP sensor, which ensures the FP cavity to be open. The open cavity makes the sensor has lower temperature-pressure cross sensitivity and the other fewer shortcoming such as sensor aging or high-temperature-induced failure because of gluing. The all-silica-glass material mitigates the shortcomings caused by the differences in the thermal expansion of materials. The stability system of the working point with an ASE light source and a FP tunable fiber filter can promote the better application of sensors. Based on the characteristics of the silica optical fiber, all-silica glass material was used to construct a FP interference optical-fiber acoustic wave sensor based on the deformation of a pressure-sensitive diaphragm. To melt the silica glass, it was irradiated with infrared light, and a CO_2_ laser was used to realize laser welding of a sensor probe. An optical path and CO_2_ laser welding process were also designed. The structural parameters of the sensor probe were optimized, and the exhaust hole of a FP cavity was reserved during laser welding. To lay a foundation for reducing the temperature–sound pressure cross sensitivity and working-point drift, the stability of the working point was analyzed, and an ASE light source and tunable fiber FP filter were proposed for stabilizing the working-point. A 40 kHz acoustic signal source was used for testing. The results show that the method can effectively detect 0.02–0.1 W/cm^2^ acoustic signal sources with a sensitivity of 6.59 mV/kPa and a linearity coefficient of 0.99975, indicating good linearity.

## Data Availability

The data are available on request from the corresponding author.
